# Flexible Coordination of Flexible Limbs: Decentralized Control Scheme for Inter- and Intra-Limb Coordination in Brittle Stars' Locomotion

**DOI:** 10.3389/fnbot.2019.00104

**Published:** 2019-12-13

**Authors:** Takeshi Kano, Daichi Kanauchi, Tatsuya Ono, Hitoshi Aonuma, Akio Ishiguro

**Affiliations:** ^1^Research Institute of Electrical Communication, Tohoku University, Sendai, Japan; ^2^Research Center of Mathematics for Social Creativity, Research Institute for Electronic Science, Hokkaido University, Sapporo, Japan

**Keywords:** brittle star, decentralized control, inter-limb coordination, intra-limb coordination, resilience

## Abstract

Conventional mobile robots have difficulties adapting to unpredictable environments or performing adequately after undergoing physical damages in realtime operation, unlike animals. We address this issue by focusing on brittle stars, an echinoderm related to starfish. Most brittle stars have five flexible arms, and they can coordinate among the arms (i.e., inter-arm coordination) as well as the many bodily degrees of freedom within each arm (i.e., intra-arm coordination). They can move in unpredictable environments while promptly adapting to those, and to their own physical damages (e.g., arm amputation). Our previous work focused on the inter-arm coordination by studying trimmed-arm brittle stars. Herein, we extend our previous work and propose a decentralized control mechanism that enables coupling between the inter-arm and intra-arm coordination. We demonstrate via simulations and real-world experiments with a brittle star-like robot that the behavior of brittle stars when they are intact and undergoing shortening or amputation of arms can be replicated.

## 1. Introduction

Modern mobile robots are required to perform adequately in harsh environments such as disaster areas, distant planets, and deep oceans (Murphy, 2004; Antonelli et al., 2008; Sanderson, 2010; Nagatani et al., 2013; Patané, 2019). The challenge now is how to make the robots coordinate, in real-time, their numerous bodily degrees of freedom under unpredictable circumstances, including changes in the environment and unexpected physical damages to the robots' structure. Previous studies tackled this problem by using learning techniques (Bongard et al., 2006; Mahdavi and Bentley, 2006; Mostafa et al., 2010; Koos et al., 2013; Christensen et al., 2014; Ren et al., 2014; Rubio et al., 2018, 2019; Yen et al., 2018) and trial-and-error methods (Cully et al., 2015), however, the performance level of robots using these techniques is not satisfactory. Specifically, the previous robots could only adapt to predictable circumstances or required a considerably long adaptation time.

Drawing inspiration from animals could be one solution to the aforementioned problem. Indeed, animals, even primitive living organisms, do not lose their functionality under unstructured and unpredictable real-world constraints, and they can adapt to various environments in real-time by coordinating their bodily degrees of freedom (Takamatsu et al., 2001; Schilling et al., 2013). This ability has been honed through evolutionary selection pressure, and it is likely that there is a sophisticated underlying mechanism. Owing to this, engineers have started implementing animal adaptation mechanisms in robots (Ijspeert, 2014).

**Figure 1:**
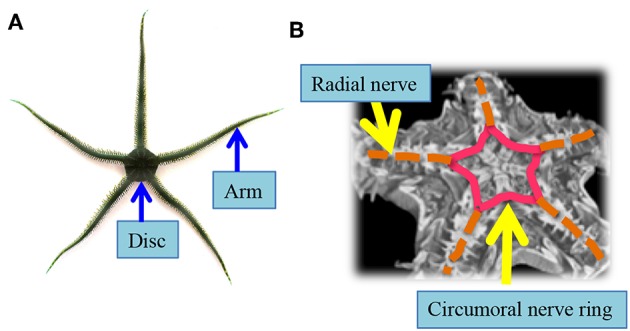
Body and nerve structure of a real brittle star (*Ophiarachna incrassata*). **(A)** Overview of a brittle star. Five flexible arms radiate from a central disc. **(B)** Micro-computed tomography image of a brittle star. The nervous system is indicated by pink and orange lines. Radial nerves that innervate the arms (orange lines) are connected via a circumoral nerve ring located in the central disc (pink lines).

Among the various animal species, in this paper, we focus on the locomotion of a brittle star; a variety in the phylum Echinodermata, which includes other varieties like starfish, sea cucumber, sea urchin etc. (Glaser, 1907; Arshavskii et al., 1976a,b; Wilkie, 1978; Cobb and Stubbs, 1981; Skold and Rosenberg, 1996; Carnevali, 2006; Astley, 2012; Kano et al., 2012, 2017; Watanabe et al., 2012; Matsuzaka et al., 2017; Clark et al., 2019). A brittle star has a central disc and five functionally interchangeable flexible arms that diverge radially from a central disc (Figure 1A), and it can move adaptively on unpredictable and unstructured terrains (Arshavskii et al., 1976b). Moreover, it has an outstanding adaptability to bodily damage; it can move even after losing most of its arms (Arshavskii et al., 1976a; Kano et al., 2017). It achieves this highly adaptive locomotion by real-time coordination of different arms (i.e., inter-arm coordination) and the many bodily degrees of freedom within each arm (i.e., intra-arm coordination) (Arshavskii et al., 1976a,b; Astley, 2012; Kano et al., 2012, 2017; Watanabe et al., 2012; Matsuzaka et al., 2017; Clark et al., 2019). Surprisingly, these coordinations are performed via an extremely simple decentralized nervous system along the arms, which join a circumoral nerve ring (Figure 1B, Supplementary Movie) (Cobb and Stubbs, 1981). Thus, brittle stars likely implement an ingenious autonomous decentralized control mechanism that enables adaptation to unexpected circumstances through the coordination of many body parts.

Thus far, the essential control mechanism underlying the brittle stars' locomotion had stayed elusive for a long time, although several studies analyzed the locomotion patterns of brittle stars (Arshavskii et al., 1976a,b; Astley, 2012). Recently, we have addressed this issue by adopting a synthetic approach to infer essential mechanisms, by constructing phenomenological mathematical models and robots (Kano et al., 2012, 2017; Watanabe et al., 2012). Therein, we proposed a simple decentralized control model for the inter-arm coordination, based on the locomotion of brittle stars whose arms were trimmed or amputated in various ways (Kano et al., 2017). We implemented this mechanism in a brittle star-like robot and demonstrated that it can immediately adapt to damages, in one or several arms, by automatically coordinating the still responsive arms, in a way similar to real brittle stars. However, as the trimmed-arm brittle stars were intensively analyzed, our previous works focused on the way different arms are coordinated (i.e., inter-arm coordination), but not on the ways multiple bodily degrees of freedom within each arm are coordinated (i.e., intra-arm coordination). Thus, it remained unclear how brittle stars move adaptively by coupling the inter- and intra-arm coordination.

Herein, we aim to elucidate the decentralized control mechanism that couples the inter- and intra-arm coordinations in brittle stars' locomotion. Based on findings of the behavior of brittle stars, with various morphologies (various arm lengths, different numbers of arms etc.) and in different environments, we propose a decentralized control model that incorporates both inter- and intra-arm coordination mechanisms. Given that we are motivated to capture the essential mechanism rather than to strictly mimic the locomotion of real brittle stars, the proposed mechanism is simple and describes the minimal requirement of the brittle stars' locomotion. The validity of the proposed control mechanism was investigated via simulations, and with an experimental robot. The results show that the proposed mechanism, despite its simplicity, can reproduce the behavior of brittle stars to some extent.

The remainder of this paper is structured as follows. In section 2, we briefly summarize behavioral findings on brittle stars. In section 3, we propose a model of brittle star locomotion. Specifically, we present a model of the mechanical system and the decentralized control mechanism for the inter- and intra-arm coordination, which was deduced from the behavioral findings. In sections 4, 5, we demonstrate that a simulated brittle star (section 4) and an experimental brittle star-like robot (section 5) reproduce the locomotion of real brittle stars. Finally, we draw our conclusions and indicate the scope of future work in section 6.

## 2. Behavioral Findings

**Figure 2:**
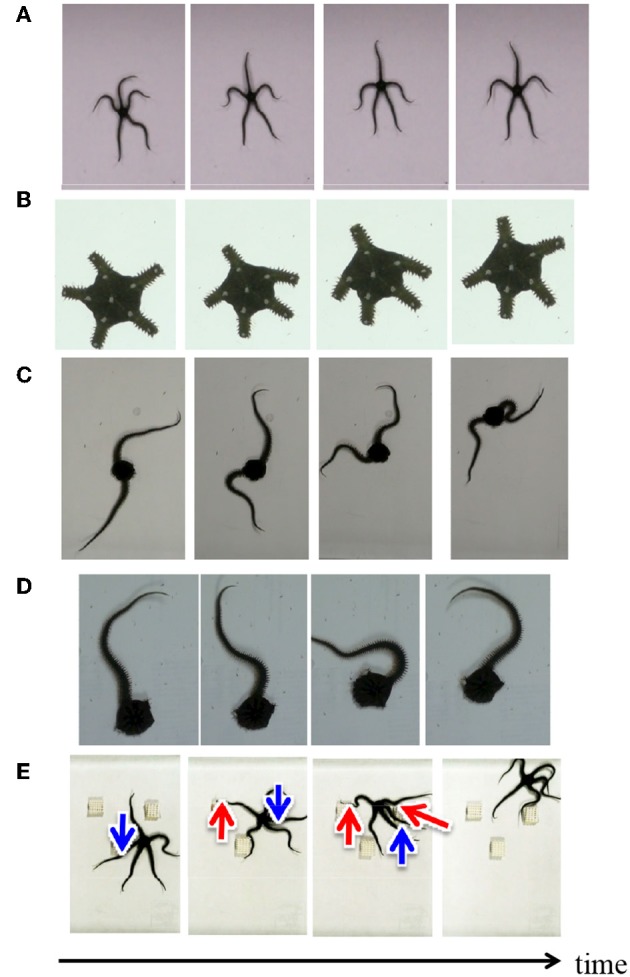
Snapshots of locomotion of real brittle stars under various situations: **(A)** an intact brittle star on a flat terrain, **(B)** a brittle star with five shortened arms on a flat terrain, **(C)** a brittle star with two arms on a flat terrain, **(D)** a brittle star with one arm on a flat terrain, and **(E)** an intact brittle star on a terrain with several square objects. Red and blue arrows denote points where the arms exploit and avoid objects, respectively.

Herein we introduce locomotion patterns of real brittle stars (*Ophiarachna incrassata*) under various conditions. Figure 2A shows a typical locomotion pattern of an intact brittle star, which is called “breaststroke” (Arshavskii et al., 1976a). The brittle star assigns distinct roles to the arms. One arm is designated the center limb, another two are the forelimbs, and there are two hindlimbs. The center limb is pointing into the movement direction. The forelimbs are the primary structures that work in coordination to move the organism forward, and the hindlimbs have a minimal role in propulsion.

Figures 2B–E showed the snapshots of the locomotion of brittle stars when the number of arms, arm length, and environment were changed in various ways (Supplementary Movie). When all the arms were shortened, the inter-arm coordination pattern was similar to the “breaststroke” of intact brittle stars (Figure 2B) (Kano et al., 2017). When some of the arms were removed, brittle stars moved by coordinating the bodily degrees of freedom in the remaining arms (Figures 2C,D). More specifically, the arms were often anchored to the ground and then they push themselves against the anchored points and effectively move. Figure 2E shows the locomotion of a brittle star on a terrain with several square objects. The arms push themselves against objects when they receive reaction forces that assist propulsion; meanwhile, they avoid the objects when they receive reaction forces that hinder propulsion.

**Figure 3:**
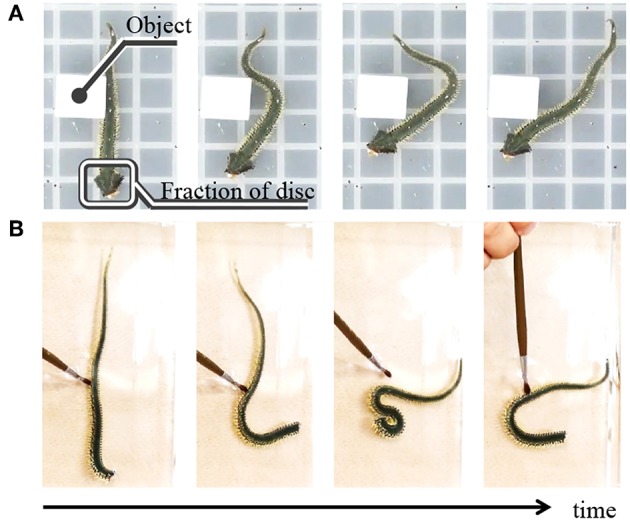
Snapshots of motion of real brittle stars when only fraction of the body remains. **(A)** Locomotion of a brittle star with one arm and its proximal end of the disc on a terrain with a square object. **(B)** Motion of an arm without the disc. The arm was physically stimulated with an ink brush to induce motion.

We also performed the following behavioral experiments to specify the origin of the inter- and intra-arm coordinations (Figure 3, Supplementary Movie). Figure 3A shows snapshots when a brittle star, with only one arm and its proximal end of the disc, is placed on a terrain with a square object. Although only a fraction of the disc remained, the arm could move by exploiting the object; thus, locomotion was observed. Figure 3B shows the snapshots of the behavior of an arm, which was completely detached from the disc. In this case, the arm could not locomote by coordinating its body parts, although it responded to physical stimuli in a reflexive manner. These results suggest that the proximal ends of the arms in the central disk play important roles in locomotion through coordination of the body parts.

## 3. Model

Based on the findings described in section 2, we propose a minimal model of the body and the control system of a brittle star, aiming to capture the essence of the inter- and intra-arm coordination. We note that the model described in this section is used for simulations in section 4. The robot slightly differs from this model, due to technical reasons, as we will describe in section 5.

### 3.1. Body

**Figure 4:**
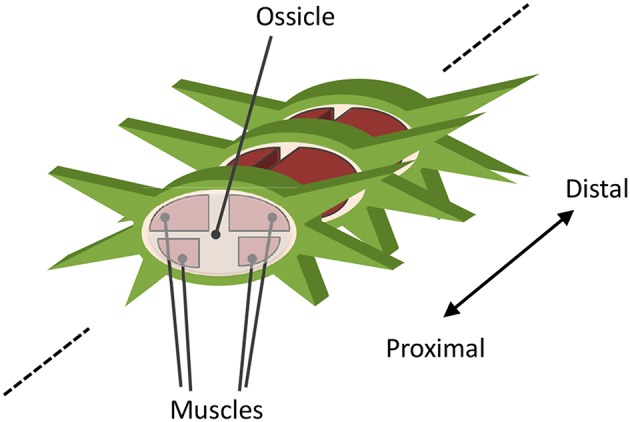
Schematic for anatomical structure of an arm.

A real brittle star comprises a central disc and five flexible arms. The arm comprises a series of segments, each containing a roughly discoidal vertebral ossicle surrounded by four muscles that connect adjacent ossicles (Figure 4) (Wilkie, 1978). The arm can bend horizontally as well as vertically by contracting these muscles. In a typical locomotion, horizontal movement is larger than that of vertical. However, vertical movement plays an important role in determining the points of ground contact.

**Figure 5:**
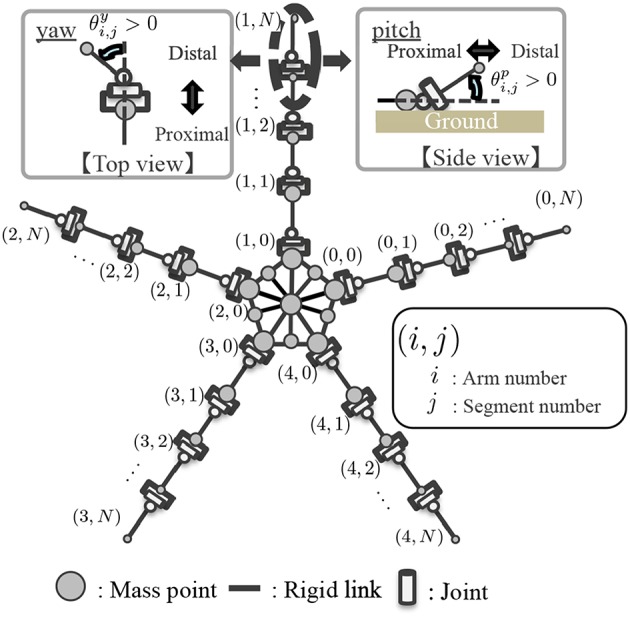
Schematic of the body model. Definition of the joint angle is shown in the magnified view.

Based on the above anatomical feature, the body is modeled as shown in Figure 5. The body is described by mass points, rigid links that connect mass points, and joints. The central disc forms a pentagon, and arms radiate from its apexes. In each arm, while intact, *N* mass points are concatenated in a one-dimensional array. Yaw and pitch joints are implemented between adjacent mass points. The arms are actuated by changing the target angles of these joints. The arms are enumerated by *i*, and the joints within each arm are enumerated by *j*. Considering that the distal side of real brittle stars' arms are thinner than the proximal side, the mass of the mass points in the arm *m*_*i,j*_ is set to decrease as *j* increases. Specifically, *m*_*i,j*_ is given by:

(1) $$\begin{array}{l} m_{i,j}=\alpha^{j}m_{\text{arm}}, \end{array}$$

where *m*_arm_ and α are positive constants, where α is smaller than 1. The mass of the mass points in the disc is set to be a constant, denoted by *m*_body_.

### 3.2. Arm Control Model

**Figure 6:**
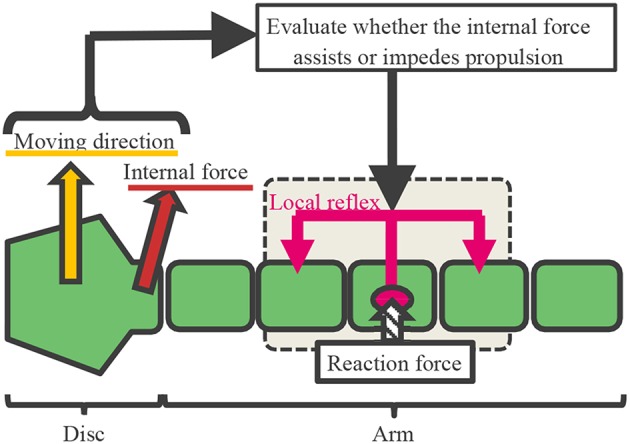
A conceptual diagram of the proposed control mechanism.

**Figure 7:**
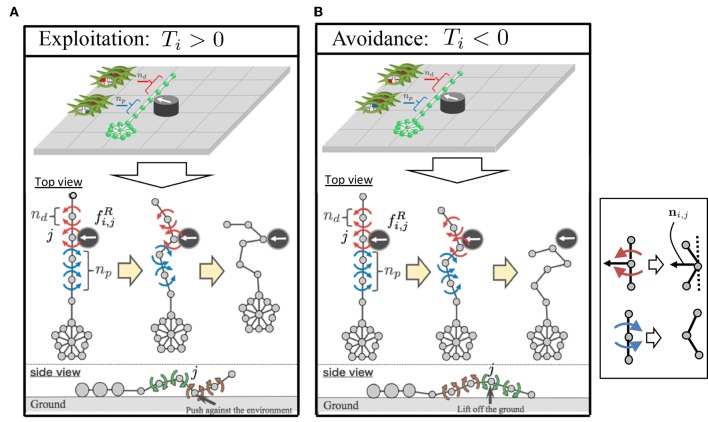
Feedback mechanism: **(A)** exploitation and **(B)** avoidance. In the upper figures, muscles that contract due to the local reflexive mechanism are depicted in red and blue for the distal and proximal segments, respectively. This local reflexive mechanism was implemented in the proposed body model (Figure 5), as shown in the lower figures. The blue and red arrows represent the torques to bend the joints, as shown in the inset. The definition of the unit vector **n**_*i,j*_ is also shown in the inset.

Based on the findings in section 2, we hypothesized that the arms are controlled according to the following procedure (Figures 6, 7 and Supplementary Movie):

1. Determining the moving direction: Although real brittle stars likely determine it by integrating stimuli received over the entire body, herein, we assume for simplicity (Figure 6) that it is given by a central command.
2. The internal force at the proximal end of each arm (namely, the force to which each arm pulls/pushes the central disc) is detected. Then, it is evaluated whether the detected force assists propulsion or not, and this information is sent to a lower level (i.e., peripheral side of the arms) (Figure 6).
3. The following local reflexive mechanisms work at the lower level, based on the result evaluated in 2).
  - When the force detected at the proximal end assists propulsion, we deduced that the following local reflexive mechanism works in real brittle stars: when a certain part of the arm receives a reaction force from the environment, several contralateral upper muscles on the distal side and ipsilateral lower muscles on the proximal side contract (Figure 7A). The muscle contraction of the distal segments works to increase the reaction force because it enables the arm to further push against the ground. Meanwhile, the muscle contraction of the proximal segments works to pull the central disc toward the contact point. Thus, the arm can exploit the environment to move effectively. This local reflexive mechanism can be expressed in the body model, as shown in Figure 5: When the arm receives a reaction force from the right(left)-hand side, several distal yaw segments bend to the left (right) while several proximal yaw segments bend to the right (left) (Figure 7A). At the same time, several distal pitch segments bend to push themselves against the ground while several proximal pitch segments bend to lift off the ground (Figure 7A).
  - When the detected force at the proximal end impedes propulsion, we deduced that the following local reflexive mechanism works in real brittle stars: when a certain part of the arm receives a reaction force from the environment, several contralateral lower muscles on the distal side and ipsilateral upper muscles on the proximal side contract (Figure 7B). The muscle contraction of the proximal segments works to make the proximal part anchored to the ground. Meanwhile, the muscle contraction of the distal segments works to raise the arm and to carry it to the moving direction. Thus, the arm can move by reducing the resistive reaction force. This local reflexive mechanism can be expressed in the body model shown in Figure 5: When the arm receives a reaction force from the right(left)-hand side, several distal yaw segments bend to the left (right) while several proximal yaw segments bend to the right (left) (Figure 7B). At the same time, several distal pitch segments bend to lift off the ground while several proximal pitch segments bend to push themselves against the ground (Figure 7B).

The above-mentioned control mechanism is mathematically described as follows. The moving direction, which is assumed to be given as a central command, is denoted by the vector **D**. Note that the absolute value |**D**| denotes the magnitude of the “will" of locomotion. The internal force at the proximal end of the *i*th arm is denoted by **F**_*i*_. Subsequently, the inner product of **D** and **F**_*i*_, denoted by *T*_*i*_, is derived. Namely,

(2) $$\begin{array}{l} T_{i}=\text{D}\cdot {\text{F}}_{i}. \end{array}$$

The detected force assists propulsion when *T*_*i*_ is positive, meanwhile, it impedes propulsion when *T*_*i*_ is negative. Then, the value of *T*_*i*_ is sent to the lower level.

The torque generated by the yaw and pitch joints, $\tau_{i,j}^{y}$ and $\tau_{i,j}^{p}$, are determined according to the proportional-derivative (PD) control (Rubio, 2016, 2018; Sun et al., 2018), namely,

(3) $$\begin{array}{l} \tau_{i,j}^{y}=\beta^{j}\left\{-k_{y}\left(\theta_{i,j}^{y}-{\bar{\theta }}_{i,j}^{y}\right)-c_{y}{\dot{\theta }}_{i,j}^{y}\right\}, \end{array}$$

(4) $$\begin{array}{l} \tau_{i,j}^{p}=\beta^{j}\left\{-k_{p}\left(\theta_{i,j}^{p}-{\bar{\theta }}_{i,j}^{p}\right)-c_{p}{\dot{\theta }}_{i,j}^{y}\right\}, \end{array}$$

where $\theta_{i,j}^{y}$ and $\theta_{i,j}^{p}$ are the real yaw and pitch joint angles, and ${\bar{\theta }}_{i,j}^{y}$ and ${\bar{\theta }}_{i,j}^{p}$ are the target yaw and pitch joint angles, *k*_*y*_ and *k*_*p*_ are the proportional gains for the yaw and pitch joints, *c*_*y*_ and *c*_*p*_ are the derivative gains for the yaw and pitch joints, respectively. The parameter β is a constant satisfying 0 < β < 1, which were introduced so that the torque generated at the distal side becomes smaller than that at the proximal side, like real brittle stars. The target joint angles are determined according to the local reflexive mechanisms described in 3(a) and (b). Thus, $\bar{\theta_{i,j}^{y}}$ and $\bar{\theta_{i,j}^{p}}$ are given by the following differential equations:

(5) $$\begin{array}{l} \tau {\dot{\bar{\theta }}}_{i,j}^{y}=-{\bar{\theta }}_{i,j}^{y}+\xi^{y}+\sigma^{y}\tanh\left(\gamma |T_{i}|S_{i,j}^{y}\right), \end{array}$$

(6) $$\begin{array}{l} \tau {\dot{\bar{\theta }}}_{i,j}^{p}=-{\bar{\theta }}_{i,j}^{p}+\xi^{p}+\sigma^{p}\tanh\left(\gamma T_{i}S_{i,j}^{p}\right), \end{array}$$

where τ, σ^*y*^, σ^*p*^, and γ are positive constants; ξ^*y*^ and ξ^*p*^ denote the noise, which are implemented to actively interact with the environment. The third terms on the right-hand side of Equations (5) and (6) denote the local reflexive mechanisms, where $S_{i,j}^{y}$ and $S_{i,j}^{p}$ are given by:

(7) $$\begin{array}{l} S_{i,j}^{y}=\overset{j}{\underset{s=\max\left\{0,j-n_{d}\right\}}{\sum }}\left(f_{i,s}^{R}-f_{i,s}^{L}\right)-\overset{\min\left\{N,j+n_{p}\right\}}{\underset{s=j+1}{\sum }}\left(f_{i,s}^{R}-f_{i,s}^{L}\right), \end{array}$$

(8) $$\begin{array}{l} S_{i,j}^{p}=\overset{j}{\underset{s=\max\left\{0,j-n_{d}\right\}}{\sum }}\left(f_{i,s}^{R}+f_{i,s}^{L}\right)-\overset{\min\left\{N,j+n_{p}\right\}}{\underset{s=j+1}{\sum }}\left(f_{i,s}^{R}+f_{i,s}^{L}\right), \end{array}$$

where *n*_*d*_ and *n*_*p*_ respectively denote the number of distal and proximal segments to which the detected force is fed back; $f_{i,j}^{R}$ and $f_{i,j}^{L}$ are defined as:

(9) $$\begin{array}{l} f_{i,j}^{R}=\max\left\{{\text{f}}_{i,j}\cdot {\text{n}}_{i,j},0\right\}\\ f_{i,j}^{L}=\max\left\{-{\text{f}}_{i,j}\cdot {\text{n}}_{i,j},0\right\} \end{array}$$

where **f**_*i,j*_ denotes the force vector acting to the *j*th mass point in the *i*th arm, and **n**_*i,j*_ is the unit vector perpendicular to the arm (Figure 7). Note that the max and min functions were introduced in Equations (7) and (8) so that the indexes do not go out of the allowed range. Thus, the feedback mechanisms described in 3(a) and (b) work when *T*_*i*_ is positive and negative, respectively.

Note that the proposed control mechanism is an extension of the control mechanism for inter-arm coordination we previously proposed (Kano et al., 2017). In fact, when each arm is shortened, the feedback to the proximal side operates in a similar manner as the local reflexive mechanism proposed in our previous work (Kano et al., 2017). Therefore, it is expected that the proposed control mechanism will enable inter-arm coordination. Additionally, the proposed control mechanism also enables the bending of long flexible arms in an appropriate manner, and thus, it is expected that intra-arm coordination can be achieved.

Given that the proposed control mechanism is based on self-organization, the theoretical analysis of the model is a challenge. Likewise, predicting its resulting behavior is also difficult. However, in the following sections, we will demonstrate the effectiveness of the proposed control mechanism and the manner in which this mechanism can reproduce the brittle stars' locomotion to a certain extent by utilizing the self-organization principle.

## 4. Simulation

**Figure 8:**
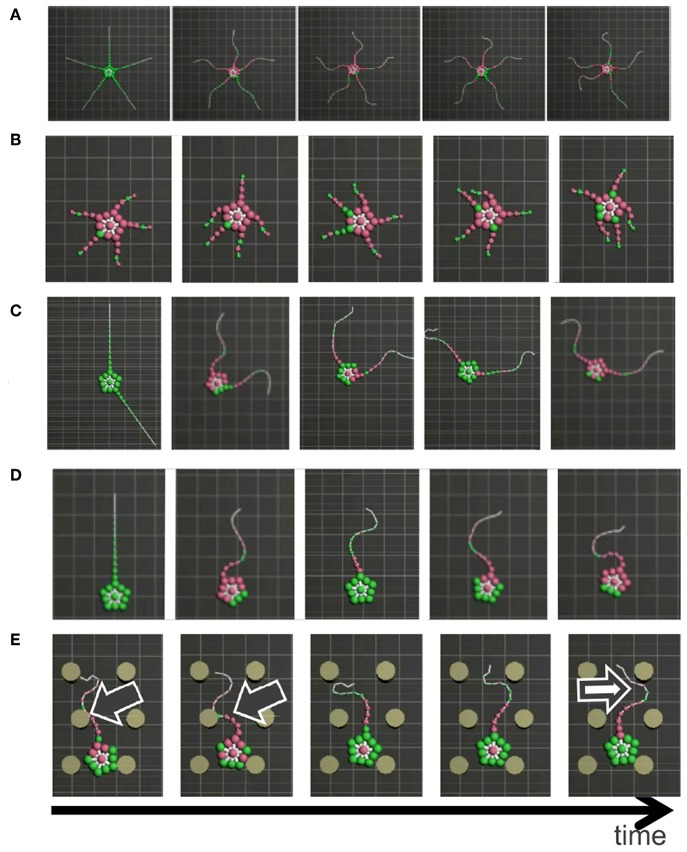
Snapshots of the simulated brittle star: **(A)** an intact brittle star on a flat terrain, **(B)** a brittle star with five shortened arms on a flat terrain, **(C)** a brittle star with two arms on a flat terrain, **(D)** a brittle star with one arm on a flat terrain, and **(E)** a brittle star with one arm on a flat terrain with several circular objects. Black and white arrows denote points where the arms exploit and avoid objects, respectively. Mass points on and off the ground are colored by green and purple, respectively.

We performed simulation experiments to validate the proposed model. The simulation source code is provided as Data Sheet 1 in the Supplementary Material. In order to simplify the calculation; the three-dimensional dynamics of the body was derived by assuming that the yaw and pitch axis of the joints were perpendicular and parallel to the ground. The vector **D** which expresses the moving direction, was set to be a unit vector. The performance should be evaluated through the adaptability to either physical damage or environments rather than through the locomotion speed under a specific environment. Unfortunately, however, to the best of our knowledge, there is no index that can measure the adaptability, and it is difficult to quantitatively define fitness. Thus, we did not apply any optimization method but determined the parameters through trial-and-error. Ideally, common parameter values should be used for all experimental conditions. Unfortunately, they had to be individually adjusted for each condition, as shown in Table 1. Specifically, σ_*y*_ was set to be slightly smaller for the five flexible arms (Figure 8A) as compared to that in the other cases (Figures 8B–E). Doing so was necessary owing to the simplicity of the proposed model. Note that σ_*p*_ was set to be considerably smaller than σ_*y*_ because vertical arm movement is smaller than horizontal arm movement in real brittle stars. However, feedback to the pitch joints plays a significant role in locomotion, because points where the body anchors the ground are accurately determined with the use of this feedback mechanism.

**Table 1:** Parameter values employed in the simulation experiments.

**Variable**	**Dimension**	**Figure 8A**	**Figures 8B–E**
α		0.8	0.8
β		0.6	0.6
*m*_body_	[kg]	1.9 × 10^−3^	1.9 × 10^−3^
*m*_arm_	[kg]	1.3 × 10^−3^	1.3 × 10^−3^
*k*_*y*_	[kgm^2^s^−2^]	6.9	6.9
*k*_*p*_	[kgm^2^s^−2^]	5.4 × 10^1^	5.4 × 10^1^
*c*_*y*_	[kgm^2^s^−1^]	3.2 × 10^−3^	3.2 × 10^−3^
*c*_*p*_	[kgm^2^s^−1^]	3.2 × 10^−3^	3.2 × 10^−3^
σ^*p*^	[deg]	1.6 × 10^−1^	1.6 × 10^−1^
σ^*y*^	[deg]	0.8	1.2
*n*_*d*_		2	2
*n*_*p*_		4	4
γ	[kg^−2^m^−2^s^4^]	4.3 × 10^11^	4.3 × 10^11^
τ	[s]	0.4	0.4

The result is shown in Figure 8 (Supplementary Movie). In the case of five flexible limbs (Figure 8A), the arms pushed themselves against the ground to move effectively (Figure 2A). When the five arms were shortened (Figure 8B), two forelimbs adjacent to the center limb tended to move synchronously, like “breaststroke" pattern of real brittle stars (Figure 2B). When two arms (Figure 8C) or only one arm (Figure 8D) remained, arms were often anchored to the ground, and then they pushed themselves against the anchored points to move effectively, like real brittle stars (Figures 2C,D). Finally, the simulated brittle star with one flexible arm moved on terrain with pegs. Then, the arm pushed against pegs and moved effectively when it received reaction forces that assisted propulsion, while objects were avoided if it received reaction forces that hinder propulsion (Figure 8E); this agrees with the behavioral findings (Figure 2E).

In the above-mentioned behaviors, inter- and intra-arm coordination was appropriately used to adapt to various circumstances. When the five arms were shortened (Figure 8B), the simulated brittle star moved through the coordination of different arms (i.e., inter-arm coordination). Meanwhile, when one arm (Figure 8D) remained, the simulated brittle star moved through the coordination of different parts within the arm (i.e., intra-arm coordination). In other cases (Figures 8A,C,E), both inter- and intra-arm coordination were used. In summary, the simulated brittle star adapted to various circumstances by coupling the inter- and intra-arm coordination and qualitatively reproducing the locomotion of real brittle stars.

**Figure 9:**
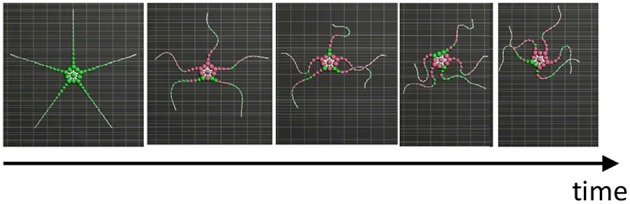
Snapshots of the simulated brittle star in the case where the arms are intact and the body is on a flat terrain with σ_*y*_ = 1.2.

As additional data, we present the result when σ_*y*_ is 1.2, which is equal to the σ_*y*_ value in Figures 8B–E (Table 1), in the case of five flexible limbs (Figure 9). The arms are observed to be more vigorous than in the case of real brittle stars. This suggests that there was an excess amount of feedback, although the parameter is suitable for the other cases (Figures 8B–E).

## 5. Robot

In this section, we present an experimental brittle star-like robot (PENTABOT III) and the results.

### 5.1. Hardware

**Figure 10:**
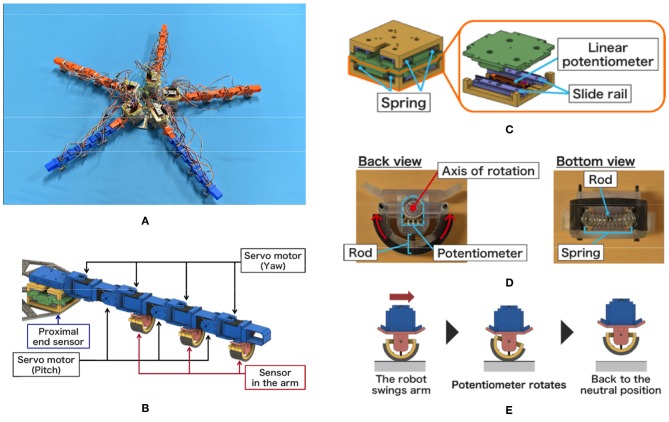
Brittle star-like robot developed. **(A)** Overview of the robot. **(B)** CAD image for the arrangement of servo motors and sensors within an arm. **(C)** Proximal end sensor. **(D)** Back view and bottom view of the sensor. **(E)** Sensing mechanism.

The overview of the robot is shown in Figure 10A. The robot consists of a central disc with five arms. The diameter of the central disc, the arm length, and the total weight was 0.22 m, 0.35 m, and 3.2 kg, respectively. Four yaw and three pitch joints were embedded alternatively in each arm, and they were driven by servo motors (Futaba Co., RS-303MR) (Figure 10B). The position control, instead of the torque control, was adopted for controlling the motor; thus, the target angles ${\bar{\theta }}_{i,j}^{y}$ and ${\bar{\theta }}_{i,j}^{p}$ were determined according to Equations (5) and (6), and we did not use Equations (3) and (4), which are used to determine the joint torques. While the mass and the gain of the actuation torque of the distal segments were smaller than those of the proximal segments in the simulation model, they were identical in the developed robot. In each arm, a microcomputer (STMicroelectronics : NUCLEO-L432KC) and a control circuit board was embedded to determine the moving direction, to detect sensor values, and to determine the target angles of the motors.

Figure 10C shows the sensor mechanism for detecting internal forces at the proximal ends of the arms. Two combinations of slide rails, springs, and a linear potentiometer (Alps Alpine Co. Ltd., RDC10320RB) were layered perpendicularly. Because it was difficult to measure the internal force directly, we simply assumed that the components of the internal force vector were proportional to the displacement of the two linear potentiometers. Thus, the sensor values of the two linear potentiometers were directly used as the components of the vector **F**_*i*_.

Figures 10D,E show the sensor mechanism detecting external forces from the environment. This mechanism consists of a semicircular rod, a potentiometer (Alps Alpine Co. Ltd., RDC506002A), and a spring. It was implemented at the bottom of servo motors that drive the yaw joints (Figure 10D). Figure 10E shows how this mechanism works. The semicircular structure at the bottom rotates when it receives external forces (e.g., frictional forces) from the right or left, and its rotational angle is measured by the potentiometer. When the arm lifts off the ground, the displacement of the potentiometer decreases due to the spring. Because it was difficult to directly measure the external force, we assumed that the external force vector was proportional to the displacement of the potentiometer for the sake of convenience. Thus, the sensor value of the linear potentiometer was directly used as the value of the external force $f_{i,j}^{R/L}$.

### 5.2. Experimental Results

We performed experiments using the experimental robot. Table 2 shows the parameter values, which were individually adjusted by trial-and-error for each body configuration shown in Figures 2A–D. The frictional property of the floor was also chosen by trial-and-error so that the robot successfully moves.

**Table 2:** Parameter values used for the experimental robot.

**Variable**	**Dimension**	**Figure 11A**	**Figure 11B**	**Figure 11C**	**Figure 11D**
σ^*y*^	[deg]	45.0	55.0	45.0	45.0
σ^*p*^	[deg]	25.0	25.0	25.0	25.0
*n*_*d*_		3	3	3	3
*n*_*p*_		3	3	3	3
γ		1.0 × 10^3^	1.0 × 10^3^	1.0 × 10^3^	1.0 × 10^3^
τ	[s]	6.0 × 10^−2^	6.0 × 10^−2^	6.0 × 10^−2^	0.15

**Figure 11:**
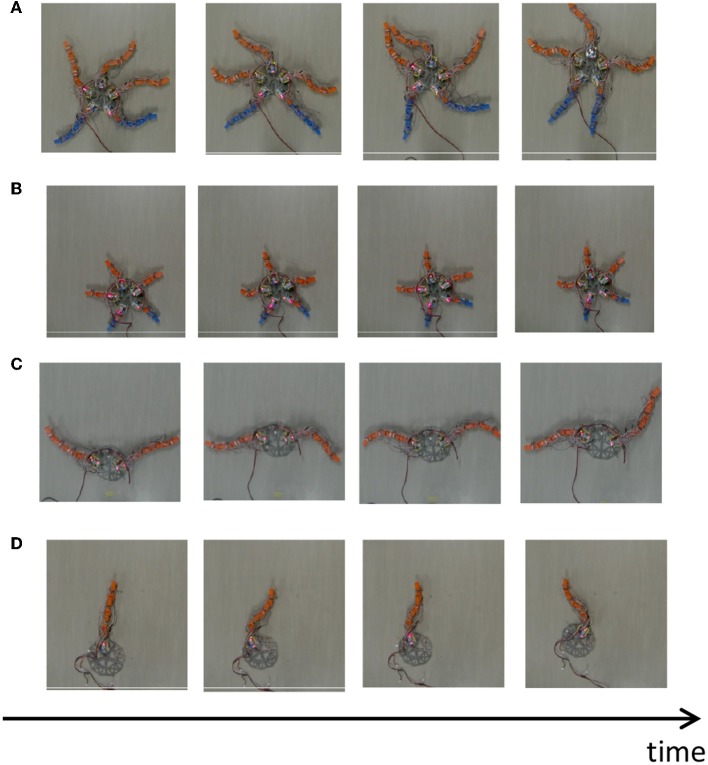
Snapshots of the brittle star-like robot **(A)** with five flexible arms, **(B)** with five shortened arms, **(C)** with two flexible arms, and **(D)** with one flexible arm. All experiments were performed on a flat terrain.

The result is shown in Figure 11 (Supplementary Movie). When all segments are present (Figure 11A), the arms pushed themselves against the ground to move effectively, which is qualitatively similar to real brittle stars (Figure 11A). When all arms were shortened (Figure 11B), the robot moved in a manner similar to a “breaststroke" pattern of real brittle stars (Figure 2B). When two arms remained (Figure 11C), the arms were often anchored to the ground, and then they pushed themselves against the anchored points to move effectively, like with real brittle stars (Figure 2C). When only one arm remained (Figure 11D), the robot moved in a qualitatively similar manner as real brittle stars (Figure 2D) although the central disc did not anchor well to the ground and was often pushed backward during arm extension. Thus, although there existed a slight discrepancy between the robot and real brittle stars, the behavioral findings were qualitatively reproduced.

## 6. Conclusion and Future Work

We focused on the locomotion of brittle stars that move by coordinating their five flexible arms. Based on behavioral findings of brittle stars with various morphologies in various environments, we proposed a simple decentralized control model that incorporates both inter- and intra-arm coordination mechanisms. We demonstrated, via simulations, that the proposed model reproduces the behavioral findings qualitatively. Moreover, we developed a brittle star-like robot and performed real-world experiments; the robot moved in a qualitatively similar manner as the real brittle stars.

Previous studies that used learning or trial-and-error techniques (Bongard et al., 2006; Mahdavi and Bentley, 2006; Mostafa et al., 2010; Koos et al., 2013; Christensen et al., 2014; Ren et al., 2014; Cully et al., 2015; Rubio et al., 2018, 2019; Yen et al., 2018) required a considerable amount of time (more than several tens of seconds) to respond to unexpected physical damage. Meanwhile, we have recently developed a brittle star-like robot that can immediately adapt to unexpected physical damage (Kano et al., 2017), yet the number of degrees of freedom within the body was still small. In contrast, this study succeeded in considerably increasing the number of bodily degrees of freedom since our previous work (Kano et al., 2017), thereby paving the way to developing robots that can coordinate a large number of bodily degrees of freedom adapting to unpredictable circumstances in real-time.

This study is also significant from a scientific viewpoint because we succeeded in capturing the essence of the inter- and intra-arm coordination mechanism in brittle stars. Moreover, we believe that our finding imparts novel insights into the essential mechanism of animals' adaptive locomotion from a general perspective. In fact, the proposed mechanism has things in common with other animals. For example, in insect locomotion, local positive feedback mechanism works depending on whether the leg supports locomotion or not (Schmitz et al., 2008), which is similar to the control mechanism proposed in this study.

However, there are limitations in this study. First, we had to fine-tune parameters for each body configuration as well as to carefully choose frictional property of the floor. Second, the robot did not move as effectively as real brittle stars. In particular, the locomotion of the robot with only one arm was extremely slow. Third, we could not reproduce locomotion on a terrain with several objects (Figure 2E) with the robot. These limitations originate from mechanical and control issues. Concerning mechanical aspects, the reaction force was not properly measured by the current sensor system used. Additionally, the mass distribution of the robot and the friction between the body and the ground were not optimal. Regarding control, the proposed control scheme is not able to fully mimic the brittle stars' locomotion owing to its simplicity, even though it likely captures the essence of the locomotion. More complex control schemes may improve performance. Solving these issues remain as future work.

Another future direction of this work is the realization of a fully autonomous brittle star-like robot. For this, from the viewpoint of mechanics, the robot must contain batteries. From the viewpoint of control, the moving direction needs to be automatically determined. In our previous works, we performed behavioral experiments wherein the nerve ring was partially damaged (Clark et al., 2019), and based on this, we proposed a mathematical model for the nerve ring and succeeded in determining the moving direction in a self-organized manner (Kano et al., 2019). We believe that the control scheme for the fully autonomous brittle star-like robot can be developed by combining the model proposed in this paper with that for the nerve ring (Kano et al., 2019).

## Data Availability Statement

The raw data supporting the conclusions of this manuscript will be made available by the authors, without undue reservation, to any qualified researcher.

## Author Contributions

TK and AI contributed the initial conception. TK, TO, HA, and AI proposed the mathematical model. TO performed simulations. DK developed the robot and performed experiments. TK wrote the manuscript. DK, TO, HA, and AI contributed to manuscript revision.

### Conflict of Interest

The authors declare that the research was conducted in the absence of any commercial or financial relationships that could be construed as a potential conflict of interest.

## Supplementary Material

Supplementary video which summarizes the research. Micro CT images were reproduced from the supplementary video Kano et al. (2017).

Data Sheet 1 Simulation source code. It is written in C++. “Monitor.cpp” and “Monitor.hpp” were mainly written by Dr. Masahiro Shimizu of Osaka University.
